# An interaction map of circulating metabolites, immune gene networks, and their genetic regulation

**DOI:** 10.1186/s13059-017-1279-y

**Published:** 2017-08-01

**Authors:** Artika P. Nath, Scott C. Ritchie, Sean G. Byars, Liam G. Fearnley, Aki S. Havulinna, Anni Joensuu, Antti J. Kangas, Pasi Soininen, Annika Wennerström, Lili Milani, Andres Metspalu, Satu Männistö, Peter Würtz, Johannes Kettunen, Emma Raitoharju, Mika Kähönen, Markus Juonala, Aarno Palotie, Mika Ala-Korpela, Samuli Ripatti, Terho Lehtimäki, Gad Abraham, Olli Raitakari, Veikko Salomaa, Markus Perola, Michael Inouye

**Affiliations:** 1 Department of Microbiology and Immunology, The University of Melbourne, Parkville, 3010 Victoria Australia; 2 Systems Genomics Lab, Baker Heart and Diabetes Institute, Melbourne, Victoria Australia; 3 Department of Pathology, The University of Melbourne, Parkville, 3010 Victoria Australia; 4 School of BioSciences, The University of Melbourne, Parkville, 3010 Victoria Australia; 5 National Institute for Health and Welfare, Helsinki, 00271 Finland; 6 Institute for Molecular Medicine Finland, University of Helsinki, Helsinki, 00014 Finland; 7 Computational Medicine, Faculty of Medicine, University of Oulu, Oulu, 90014 Finland; 8 NMR Metabolomics Laboratory, School of Pharmacy, University of Eastern Finland, Kuopio, 70211 Finland; 9 University of Tartu, Estonian Genome Center, Tartu, 51010 Estonia; 10 Diabetes and Obesity Research Program, University of Helsinki, Helsinki, Finland; 11 Biocenter Oulu, University of Oulu, Oulu, 90014 Finland; 12 Department of Clinical Chemistry, Fimlab Laboratories and Finnish Cardiovascular Research Center-Tampere, Faculty of Medicine and Life Sciences, University of Tampere, 33014 Tampere, Finland; 13 Department of Clinical Physiology, University of Tampere and Tampere University Hospital, FI-33521 Tampere, Finland; 14 Department of Medicine, University of Turku and Division of Medicine, Turku University Hospital, FI-20520 Turku, Finland; 15 Murdoch Childrens Research Institute, Parkville, 3052 Victoria Australia; 16 Analytic and Translational Genetics Unit, Department of Medicine, Massachusetts General Hospital, Boston, Massachusetts USA; 17 Program in Medical and Population Genetics, Broad Institute of Harvard and MIT, Cambridge, Massachusetts USA; 18 Psychiatric & Neurodevelopmental Genetics Unit, Department of Psychiatry, Massachusetts General Hospital, Boston, Massachusetts USA; 19 Computational Medicine, School of Social and Community Medicine, University of Bristol, Bristol, BS8 1TH UK; 20 Medical Research Council Integrative Epidemiology Unit, University of Bristol, Bristol, BS8 2BN UK; 21 Department of Public Health, University of Helsinki, Helsinki, 00014 Finland; 22 Department of Clinical Physiology and Nuclear Medicine, Turku University Hospital, Turku, 20520 Finland; 23 Research Centre of Applied and Preventive Cardiovascular Medicine, University of Turku, Turku, 20520 Finland

## Abstract

**Background:**

Immunometabolism plays a central role in many cardiometabolic diseases. However, a robust map of immune-related gene networks in circulating human cells, their interactions with metabolites, and their genetic control is still lacking. Here, we integrate blood transcriptomic, metabolomic, and genomic profiles from two population-based cohorts (total N = 2168), including a subset of individuals with matched multi-omic data at 7-year follow-up.

**Results:**

We identify topologically replicable gene networks enriched for diverse immune functions including cytotoxicity, viral response, B cell, platelet, neutrophil, and mast cell/basophil activity. These immune gene modules show complex patterns of association with 158 circulating metabolites, including lipoprotein subclasses, lipids, fatty acids, amino acids, small molecules, and CRP. Genome-wide scans for module expression quantitative trait loci (mQTLs) reveal five modules with mQTLs that have both *cis* and *trans* effects. The strongest mQTL is in *ARHGEF3* (rs1354034) and affects a module enriched for platelet function, independent of platelet counts. Modules of mast cell/basophil and neutrophil function show temporally stable metabolite associations over 7-year follow-up, providing evidence that these modules and their constituent gene products may play central roles in metabolic inflammation. Furthermore, the strongest mQTL in *ARHGEF3* also displays clear temporal stability, supporting widespread *trans* effects at this locus.

**Conclusions:**

This study provides a detailed map of natural variation at the blood immunometabolic interface and its genetic basis, and may facilitate subsequent studies to explain inter-individual variation in cardiometabolic disease.

**Electronic supplementary material:**

The online version of this article (doi:10.1186/s13059-017-1279-y) contains supplementary material, which is available to authorized users.

## Background

Over the past decade increasing evidence has implicated inflammation as a probable causal factor in metabolic and cardiovascular diseases. Consequently, research has begun to focus on the interplay between immunity and metabolism, or immunometabolism. While it is involved in diverse pathophysiologies, immunometabolism is particularly relevant to diseases of immense global health burden, such as type 2 diabetes (T2D) and atherosclerosis.

For T2D, immune overactivation in adipose tissue has been implicated as a key driver [1, 2]. Studies have shown that macrophage infiltration and subsequent overexpression of proinflammatory cytokines, such as TNF-α, in adipose tissues is associated with insulin resistance [1, 2]. Moreover, evidence for metabolic inflammation has been shown in other tissues where, in blood, elevated glucose and free fatty acid levels potentiate IL-1β-mediated destruction of pancreatic ß cells and subsequent T2D progression [3–5]. While circulating metabolites are known to be associated with cardiovascular disease [6], inflammation is an increasingly recognized factor in pathogenesis. In atherosclerosis, lipid-induced inflammatory response mechanisms have also been implicated in progression to myocardial infarction [7]. In atherogenic lesions, oxidized phospholipids are known to lead to a new macrophage phenotype [8], and cholesterol loading in macrophages promotes proinflammatory cytokine secretion [9].

Perhaps surprisingly, few large-scale studies have systematically assessed interactions between the human immune system and metabolites. Recent studies have investigated matched blood transcriptomic and metabolomic profiles to understand their interplay [10–16]. However, these studies had modest sample sizes and thus have not had the power to focus on the diverse range of immune processes that interact with circulating metabolites. Furthermore, even fewer have assessed effects of expression quantitative trait loci (eQTLs) on immune gene networks. A robust integrated map of immunometabolic relationships and their genetic regulation would provide a foundation for investigating the differential cardiometabolic disease susceptibility amongst individuals while also identifying key target interactions for mechanistic in vivo and in vitro follow-up.

In this study, we present an integrated immunometabolic map using matched blood metabolomic and transcriptomic profiles from 2168 individuals from two population-based cohorts. We perform gene coexpression network discovery and cross-cohort replication to identify robust gene modules which encode immune-related functions. Using a high-throughput quantitative NMR metabolomics platform that can separate lipids and lipoprotein sub-fractions as well as quantify a panel of polar metabolites, we identify significant interactions between immune gene modules and circulating metabolite measures. Genome-wide scans for QTLs affecting immune gene modules identify many *cis* and *trans* loci affecting module expression. Finally, we test the long-term stability of gene modules, their interactions with metabolite measures, and genetic control using a 7-year follow-up sampling of 333 individuals.

## Results and discussion

### Summary of cohorts and data

**Fig. 1:**
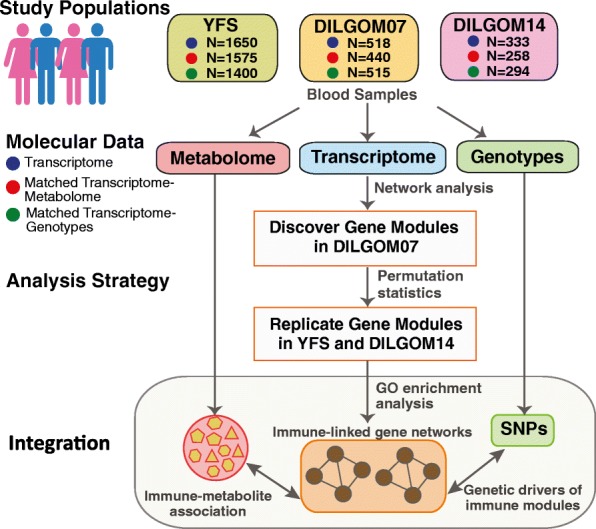
The study design. *GO* Gene Ontology, *SNP* single nucleotide polymorphism

We analyzed genome-wide genotype, whole blood transcriptomic, and serum metabolomics data from two population-based cohorts (“Methods” and Fig. 1). In DILGOM07, 240 males and 278 females aged 25–74 years were recruited (total N = 518). Data were available for a subset of 333 participants from DILGOM07 who were followed up after 7 years (DILGOM14). In YFS, relevant data were available for 755 males and 895 females aged 34–49 years (total N = 1650).

DILGOM and YFS genotyping was performed using Illumina Human 610 and 670 arrays, respectively, with subsequent genotype imputation performed using IMPUTE2 [17] and the 1000 Genomes Phase I version 3 reference panel. For both cohorts, whole blood transcriptome profiling was performed using Illumina HT-12 arrays and serum metabolomics profiling was carried out using the same serum NMR metabolomics platform (Brainshake Ltd) [18]. Individuals on lipid-lowering medication and pregnant women were excluded from the metabolome analyses (“Methods”). Of the 159 metabolite measures analyzed, 148 were directly quantified and 11 derived (Additional file 1: Table S1). After filtering, matched transcriptome and metabolome data were available for 440 individuals in DILGOM07 and 216 of these individuals (DILGOM14) who were profiled at 7-year follow-up. In YFS, 1575 individuals were available with similar data (see “Methods” for details).

### Robust immune gene coexpression networks from blood

We first identified networks of tightly coexpressing genes in DILGOM07 and then used a permutation approach, NetRep [19], to statistically test replication patterns of density and connectivity for these networks in YFS. For module detection, we applied weighted gene coexpression network analysis (WGCNA) to all 35,422 probes in the DILGOM07 data, identifying a total of 40 modules of coexpressed genes (“Methods”). For each module, we used NetRep to calculate seven preservation statistics in the YFS, generate empirical null distributions for each of these test statistics, and calculate their corresponding *P* values [19, 20]. A module was considered strongly preserved if the *P* value was <0.001 for all seven preservation statistics (Bonferroni correction for 40 modules). Of the 40 DILGOM07 modules, 20 were strongly preserved in YFS (Additional file 2: Table S2). For each of the 20 replicated modules, we defined core gene probes, those which are most tightly coexpressed and thus robust to clustering parameters, using a permutation test of module membership (“Methods”; Additional file 3: Table S3).

To identify modules of putative immune function, we carried out Gene Ontology (GO) biological process enrichment analysis using GOrilla for the core genes of each replicated module [21]. Significant GO terms (false discovery rate (FDR) <0.05) were then summarized into representative terms based on semantic similarity using REVIGO [22] (Additional file 4: Figure S1). A module was considered immune-related if it was significantly enriched for GO terms “immune system processes” (GO:0002376) and/or “regulation of immune system processes” (GO:0002682) in the REVIGO output. Six out of 20 modules were enriched for at least one of these terms (Additional file 5: Table S4). We also identified two additional modules which were not enriched for any GO terms but have been previously linked to immune functions related to mast cell and basophil function [13] and platelet aggregation activity [23]. The eight modules encoded diverse immune functions, including cytotoxic, viral response, B cell, platelet, neutrophil, mast cell/basophil, and general immune-related functions. Each immune module’s gene content and putative biological function is summarized in Table 1.

**Table 1:** Immune module gene content and putative biological function based on GO terms (top three shown) and literature

Module	Size	GO terms	Literature-based immune-related function of genes
Cytotoxic cell-like module (CCLM)	130 (115)	Immune system process Defense response Immune response	Cytotoxic effectors (*GZMA*, *GZMB*, *GZMM*, *CTSW*, *PRF1* [66]); surface receptors (*IL2RB*, *SLAMF6*, *CD8A*, *CD8B*, *CD2*, *CD247*, *KLRD1*, *KLRG1* [66–68]); T and NK cell differentiation (*ID2* and *EOMES* [69, 70]), activation (*ZAP70* and *CBLB* [71, 72]), and recruitment (*CX3CR1*, *CCL5*, *CCL4L2* [73])
Viral response module (VRM)	95 (88)	Response to virus Type I interferon signaling pathway Response to biotic stimulus	Type I interferon-induced antiviral activity (*IFITM1*, *IFIT1*, *IFIT2*, *IFIT3*, *IFIT5*, *IFI44*, *IFI44L*, *IFI6*, *MX1*, *ISG15*, *ISG20*, *HERC5* [74, 75]); viral RNA degradation (*OAS1*, *OAS2*, *OAS3*, *OASL*, *DDX60* [30]); type 1 interferon-signaling pathway (*IRF9*, *STAT1*, *STAT2* [76, 77])
B-cell activity module (BCM)	54 (49)	Immune system process Immune response B cell activation	B cell surface markers (*CD79A*, *CD79B*, *CD22* [33, 78]); B cell activation (*BANK1*, *BTLA*, *CD40*, *TNFRSF13B*, *TNFRSF13C* [79]), development (*POU2AF1*, *BCL11A*, *RASGRP3* [80]), migration (*CXCR5*, *CCR6* [80, 81]), and their regulation (*CD83*, *FCER2*, *FCRL5* [82]); antigen presentation (*HLA-DOA*, *HLA-DOB* [83])
Platelet module (PM)^a^	114 (106)	Coagulation Blood coagulation Cell activation	Platelet receptor signaling, activation, and coagulation (*GP6*, *GP9*, *ITGA2B*, *ITGB3*, *ITGB5*, *MGLL*, *MPL*, *MMRN1*, *PTK2*, *VCL*, *THBS1*, *F13A1*, *VWF*, [84]); regulating platelet activity (*SEPT5*, *TSPAN9* [85, 86])
Neutrophil module (NM)^a^	26 (26)	Killing of cells of other organism Cell killing Response to fungus	Anti-microbial, -fungal, and -viral activity (*DEFA1*, *DEFA1B*, *DEFA3*, *DEFA4*, *ELANE*, *BPI*, *RNASE2*, *RNASE3* [87–90]); neutrophil-mediated activity (*AZU1*, *LCN2*, *MPO*, *CEACAM6*, *CEACAM8*, *OLFM4* [90, 91]) and its regulation (*LCN2*, *CAMP*, *OLR1* [49, 92, 93])
Lipid-leukocyte module (LLM)^a^	13 (13)	Mast cell and basophil function^b^	Mast cell and basophil related immune response and allergic inflammation (*FCER1A*, *HDC*, *GATA2*, *SLC45A3*, *CPA3*, *MS4A3* [13, 94, 95])
General immune module A (GIMA)	509 (482)	Immune system process Defense response Regulation of response to stimulus	These modules contain genes involved in a broad range of immune processes and their regulation such as signaling; cell death; defense response to stress, inflammation, and external stimuli; leukocyte activation, migration, and adhesion
General immune module B (GIMB)	74 (69)	Immune response-activating signal transduction Positive regulation of immune response Activation of immune response	

Size refers to the number of core genes in each module and the subset of these core genes with GO term annotations are listed in parentheses. Functions were assigned to each of these modules based on GO enrichments and literature-based searches for genes in the modules.

^a^ Modules previously reported to have immune related function

^b^ The LLM was not significantly enriched for any GO term

### Immune module association analysis for eQTLs and metabolite measures

**Fig. 2:**
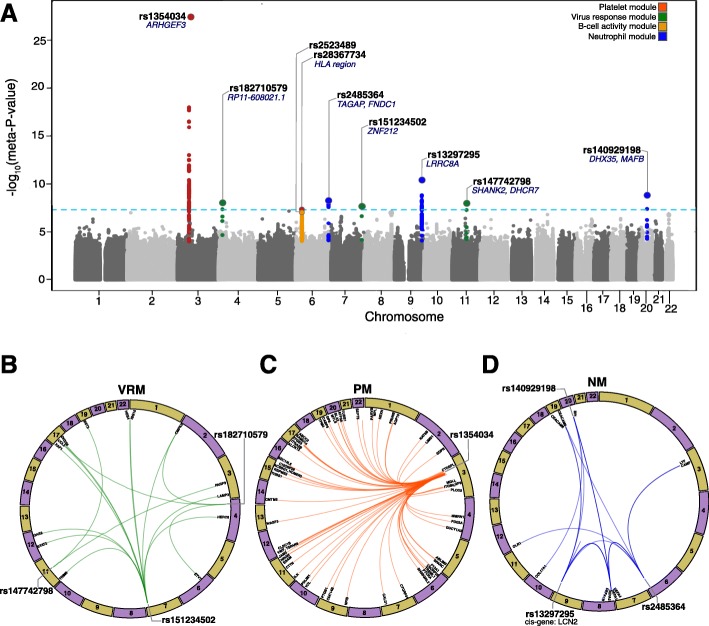
Module and expression QTL analysis. **a** Manhattan plot of meta-analyzed *P* values from the DILGOM/YFS module QTL analysis. The lead SNP and its closest genes are noted. Each significant mQTL locus is colored by module. The *horizontal dashed line* represents genome-wide (meta﻿﻿- *P* value <5 × 10^−8^) significance. **b**–**d** Circular plots summarizing the individual gene associations (meta-*P* value <5 × 10^−8^) for the lead module QTLs in the VRM, PM, and NM. Lead SNPs and *cis* genes are labeled outside the ring. *PM* platelet module, *VRM* viral response module, *CCLM* cytotoxic cell-like module, *NM* neutrophil module, *BCM* B-cell activity module

For each gene module, we performed a genome-wide scan to identify module QTLs (mQTLs) that regulate expression. In DILGOM07 and YFS, the module eigengene was regressed on each SNP, then mQTL test statistics were combined in a meta-analysis (“Methods”). Significant mQTLs were further examined at individual gene expression levels. A genome-wide significance level (*P* value <5 × 10^−8^) was used to identify mQTLs and significant *trans* effects on individual gene expression (Fig. 2 and Table 2). Leukocyte and platelet counts were available for YFS and were used to test the robustness of module associations with mQTLs and metabolite measures. Six modules showed statistically significant association with platelet or leukocyte counts (*P* value <0.05) (Additional file 6: Table S5); however, adjustment for leukocyte counts did not affect mQTL nor module-metabolite measure associations, with the exception of the platelet module (PM) and cytotoxic cell-like module (CCLM) discussed below (Additional file 7: Table S6). Since we did not have cell counts available for DILGOM07, all the associations between immune modules and metabolite measures discussed below, unless otherwise noted, have not been adjusted for cell counts.

**Table 2:** QTLs for immune gene modules

Module	Top SNP	Chr	Hg19 pos. (Mb)	Allele (minor/major)	MAF (avg)	*P* value DILGOM07 (effect size)	*P* value YFS (effect size)	Meta *P* value
VRM	rs182710579	4	19768086	G/T	0.012	2.01 × 10^–4^ (0.05)	8.10 × 10^–6^ (0.02)	9.23 × 10^–9^
	rs151234502	7	148950168	T/C	0.012	2.59 × 10^–1^ (0.01)	5.31 × 10^–9^ (0.03)	2.46 × 10^–8^
	rs147742798	11	70947761	T/C	0.016	1.51 × 10^–3^ (0.04)	1.66 × 10^–6^ (0.02)	9.43 × 10^–9^
BCM	rs2523489	6	31348878	T/C	0.186	1.42 × 10^–1^ (0.005)	5.29 × 10^–8^ (0.006)	6.27 × 10^–8^
PM	rs1354034	3	56849749	T/C	0.284	7.11 × 10^–14^ (-0.02)	1.51 × 10^–16^ (-0.008)	7.35 × 10^–28^
	rs28367734	6	3128657	A/G	0.108	5.40 × 10^–4^ (0.02)	2.02 × 10^–5^ (0.006)	5.44 × 10^–8^
NM	rs2485364	6	159512260	C/T	0.466	1.78 × 10^–3^ (0.009)	6.05 × 10^–7^ (0.004)	3.93 × 10^–9^
	rs13297295	9	131659724	C/T	0.085	4.26 × 10^–2^ (0.009)	8.39 × 10^–11^ (0.01)	3.93 × 10^–11^
	rs140929198	20	38555870	A/G	0.031	2.98 × 10^–2^ (0.03)	8.47 × 10^–9^ (0.01)	1.41 × 10^–9^
GIMA	rs2185366	8	131342722	T/C	0.421	2.0 × 10^–2^ (0.007)	1.52 × 10^–6^ (0.004)	1.05 × 10^–7^

Modules: *VRM* viral response module, *BCM* B-cell activity module, *PM* platelet module, *NM* neutrophil module, *GIMA* general immune module A. *MAF* minor allele frequency

### Cytotoxic cell-like module

**Fig. 3:**
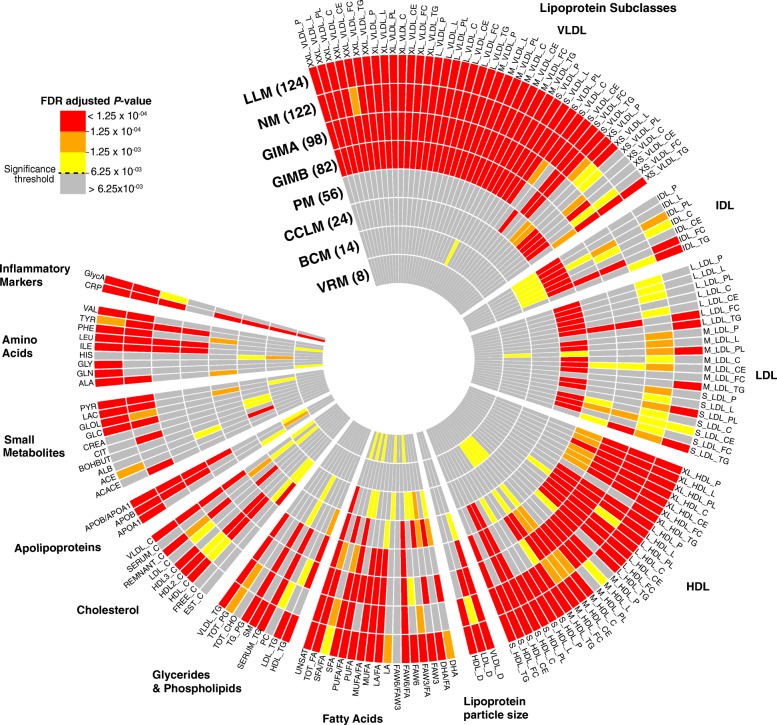
Metabolite measure associations with immune gene modules. Circular heatmap of associations between individual metabolite measure and the module eigengene of each module (colored by FDR-adjusted *P* values). Concentric circles represent modules, with numbers in parentheses denoting total number of metabolite measures associated with that module at FDR-adjusted *P* value <6.25 × 10^–3^. *NM* neutrophil module, *LLM* lipid leukocyte module, *GIMA*/*GIMB* general immune module A/B, *PM* platelet module, *CCLM* cytotoxic cell-like module, *BCM* B-cell activity module, *VRM* viral response module. See Additional file 1: Table S1 for full metabolite descriptions

CCLM was associated with 24 metabolite measures, mainly consisting of fatty acids, intermediate density lipoproteins, and C-reactive protein (CRP; Fig. 3; Additional file 8: Table S7). Of these, the average degree of unsaturation in fatty acids was the most significant association (meta-*P* value = 7.23 × 10^–7^). The immunomodulatory effects of polyunsaturated fatty acids are well characterized; for example, omega-3 fatty acids have been shown to induce cytotoxicity in in vitro cancer cell lines as well as animal models of tumor incidence and growth [24, 25]. Adjustment of the associations between CCLM and metabolite measures for leukocyte counts resulted in the gain of 38 additional associations and loss of four (creatinine, ratio of polyunsaturated fatty acids to total fatty acids, very low density lipoprotein (VLDL) particle size, and CRP) existing associations (Additional file 7: Table S6). Varying proportions of leukocyte counts can be correlated with transcription-level variation in human blood [26] but not act to confound the latter's association with phenotypes. If this is the case, then adjusting for leukocyte count in the linear regression analysis can reduce noise and thus boost statistical power to detect an association, which may explain the additional associations noted with the CCLM module. CCLM had no significant mQTLs.

### Viral response module

Three genome-wide significant mQTLs were identified for the viral response module (VRM; Fig. 2a; Table 2). The strongest mQTL, rs182710579 (meta*-P* value = 9.22 × 10^–9^), is within a known lincRNA locus (RP11-608O21.1) (Additional file 4: Figure S2a). Rs182710579 was a *trans* eQTL for three genes in the VRM (Fig. 2b; Additional file 9: Table S8). The strongest association was seen with *CCL2* (meta-*P* value = 6.78 × 10^–12^), a pro-inflammatory chemokine involved in leukocyte recruitment during viral infection [27, 28]. Also, adipocyte-derived CCL2 is known to play an important role in obesity-associated adipose tissue inflammation and insulin resistance [29]. The next strongest mQTL, rs151234502, resides within intron 4 of the relatively unstudied *ZNF212*, part of a zinc finger gene cluster at 7q36 (Additional file 4: Figure S2b). Rs151234502 modulated expression of 11 VRM genes *in trans* (Fig. 2b; Additional file 9: Table S8). The strongest association was with *OAS2* (meta-*P* value = 8.98 × 10^–10^), an interferon-induced gene encoding an enzyme promoting RNase L-mediated cleavage of viral and cellular RNA [30]. The third mQTL, rs147742798, was an intergenic SNP located between *SHANK2* and *DHCR7* at 11q13.4 (Additional file 4: Figure S2c). Rs147742798 was a *trans* eQTL for two genes in the VRM, *BST2* and *PARP9* (Fig. 2b; Additional file 9: Table S8). *BST2* encodes a trans-membrane protein with interferon-inducible antiviral function [31]. Studies have previously shown induction of fatty acid biosynthesis by a range of viruses [32]. VRM was associated with eight metabolite measures, including amino acids (alanine, phenylalanine), fatty acids (omega-6 fatty acids, polyunsaturated fatty acids, saturated fatty acids, and total fatty acids), and cholesterol esters in medium VLDL (Fig. 3; Additional file 8: Table S7). Consistent with its putative role in viral response, VRM was strongly associated with CRP (meta *P* value = 2.38 × 10^–10^).

### B-cell activity module

The B-cell activity module (BCM) was associated with 14 metabolite measures including CRP, histidine, lactate, apolipoprotiens, and mainly the medium high-density lipoprotein (HDL) subclass of lipoproteins (Fig. 3; Additional file 8: Table S7). The strongest association was seen with CRP (meta-*P* value = 2.65 × 10^–8^). Histidine was the second strongest association. This is interesting given that histidine is a substrate for histamine, and both histamine release and B-cell activity are central parts of an allergic reaction. While no mQTLs for BCM reached genome-wide significance, there was some evidence in the YFS for the MHC class I locus (Fig. 2a and Table 2). The top signal was located between *HLA-B/C* and *MICA* (rs2523489, meta-*P* value = 6.27 × 10^–8^; Additional file 4: Figure S3). The HLA class I region is well known to be associated with autoimmune diseases, where the role of B cells is well recognized. Rs2523489 was a *trans* eQTL for *CD79B* (meta-*P* value = 1.16 × 10^–9^), a subunit of the antigen-binding B-cell receptor complex [33]*.*

### Platelet module

PM had the strongest mQTL of any gene module, an intronic SNP of the *ARHGEF3* gene at 3p14.3 (rs1354034; meta-*P* value = 7.35 × 10^–28^, Fig. 2a; Table 2; Additional file 4: Figure S4a). *ARHGEF3* encodes a Rho guanine nucleotide exchange factor, a catalyst of Rho GTPase conversion from inactive GDP-bound to active GTP-bound form. Rs1354034 was an eQTL for the majority of genes in the PM, all of which were *in trans*. An intergenic SNP, rs2836773 (meta-*P* value = 5.4 × 10^–8^), at the HLA locus was also identified as an mQTL for PM (Additional file 4: Figure S4b). The *ARHGEF*3 mQTL (rs1354034) exhibited a strong *trans*-regulatory effect and was associated with 61 PM genes (65 unique probes) (Fig. 2c; Additional file 9: Table S8). The top *trans* eQTL was *ITGB3* (meta-*P* value = 5.09 × 10^–42^), a gene encoding the β_3_ subunit of the heterodimeric integrin receptor (integrin α_IIb_β_3_). This integrin receptor is most highly expressed on activated platelets and plays a key role in mediating platelet adhesion and aggregation upon binding to fibrinogen and Willebrand factor [34, 35]. Our data are consistent with previous observations of the diverse *trans* eQTL effects of rs1354034 [23], including the putative splice-QTL effects of rs1354034 on *TPM4*, a significant eGene in the PM.

*ARHGEF3* itself is of intense interest to platelet biology. It has previously been shown that silencing of *ARHGEF3* in zebrafish prevents thrombocyte formation [36]. To test whether *ARHGEF3* expression had an effect on PM genes, we regressed out *ARHGEF3* levels and re-ran the eQTL analysis. Adjusting for *ARHGEF3* did not attenuate the *trans*-associations of rs1354034, suggesting either independence of downstream function for *ARHGEF3* and rs1354034 or post-transcriptional modification of ARHGEF3. Previous GWAS studies have shown rs1354034 is associated with platelet count and mean platelet volume [36]; however, perhaps due to power, we found no significant relationship between platelet counts and rs1354034 in YFS. While platelet counts were positively associated with the PM (β = 0.29; *P* value = 8.23 × 10^–30^; Additional file 6: Table S5), the association between rs1354034 and the PM was still highly significant when conditioning on platelet counts (β = −0.33; *P* value = 1.40 × 10^–17^).

PM displayed diverse metabolic interactions and was associated with 55 metabolite measures, largely comprising of lipoprotein subclasses and fatty acids, as well as CRP (Fig. 3; Additional file 8: Table S7). Cholesterol esters in small HDL particles were most strongly associated with the PM (meta-*P* value = 9.45 × 10^–20^). HDL has been shown to exhibit antithrombotic properties by modulating platelet activation and aggregation and the coagulation pathway [37]. Also, various LDL subclasses of lipoproteins were associated with the PM, which is consistent with our understanding that LDL influences platelet activity. For example, LDL has been shown to influence platelet activity either by enhancing platelet responsiveness to aggregating stimuli or by inducing aggregation [38, 39]. Moreover, LDL-specific binding sites on platelets have also been reported [40, 41]. As noted above, the PM was associated with platelet counts, and adjustment for platelet counts in the YFS resulted in attenuation of approximately half of the weakest associations between PM and metabolite measures; however, the strongest were maintained (Additional file 7: Table S6). Association with VLDL particle size and three others were gained following the adjustment (Additional file 7: Table S6).

### Neutrophil module

Three loci were identified as mQTLs for the neutrophil module (NM; Fig. 2a and Table 2). The top mQTL was intronic to *LRRC8A* at 9q34.11 (rs13297295; meta-*P* value = 3.93 × 10^–11^; Additional file 4: Figure S5a). *LRRC8A* encodes a trans-membrane protein shown to play a role in B- and T-cell development and T cell function [42, 43]. Two additional intergenic mQTLs were located at the *TAGAP* locus at 6q25.3 (rs2485364; meta-*P* value = 3.93 × 10^–9^) and at 20q12 (rs140929198; meta-*P* value = 1.41 × 10^–9^) (Additional file 4: Figure S5b, c). Rs13297295 was a strong *trans* regulator of NM and was an eQTL for eight NM genes (ten unique probes), in particular the major alpha defensins (*DEFA1-DEFA4*), the genes of highest centrality in the module (Fig. 2d; Additional file 9: Table S8). Rs13297295 was a *cis-*eQTL for another core NM gene, *LCN2* (permuted meta-*P* value = 1 × 10^–4^) (Fig. 2d; Additional file 9: Table S8). *LCN2* is expressed in neutrophils and inducible by TLR activation, acting as an antimicrobial agent via sequestration of bacterial siderophores to prevent iron uptake [44–46]. *LCN2*’s role in acute phase response appears to be related to cardiovascular diseases, such as heart failure [47]. At the *TAGAP* locus, rs2485364 was a *trans*-eQTL for eight NM genes (ten probes) and was also a strong driver of *LCN2* (meta-*P* value = 9.11 × 10^–17^) (Fig. 2d and Additional file 9: Table S8). Consistent with our findings, neutrophils from *LCN2*-deficient mice have been shown to have impaired chemotaxis and phagocytic capability and increased susceptibility to bacterial and yeast infections compared to wild type [48, 49]. This suggests a possible functional role of *TAGAP* variants in regulating neutrophil migration through *LCN2*.

NM was associated with 121 circulating metabolite measures (~76% of all metabolite measures analyzed) as well as CRP (Fig. 3; Additional file 8: Table S7). The strongest is the previously reported association with inflammatory biomarker GlycA (meta-*P* value = 2.68 × 10^–25^) [10]; however, NM’s association with various lipoprotein subclasses, particle sizes of lipoproteins, fatty acids, cholesterol, apolipoproteins, glycerides and phospholipids, amino acids, and other small molecules indicates it has a potentially major role in linking neutrophil function to metabolism.

### Lipid-leukocyte module

Together with NM, the lipid-leukocyte module (LLM) showed extensive metabolic associations. Overall, 123 metabolite measures and CRP were associated with LLM, with the strongest being the ratio of triglycerides to phosphoglycerides (meta-*P* value = 5.16 × 10^–138^; Fig. 3; Additional file 8: Table S7). With the inclusion of the YFS, these findings strongly replicate previous associations between LLM and metabolite measures [14] as well as detecting additional associations. We also confirm the previous strong negative association between CRP and LLM (meta-*P* value = 8.16 × 10^–20^). Consistent with previous studies, no mQTLs were detected for LLM.

### General immune modules A and B

No mQTLs were associated with general immune modules A and B (GIMA and GIMB); however, these modules were associated with 97 and 82 metabolite measures, respectively (Fig. 3; Additional file 8: Table S7). Cholesterol esters in small HDL and the mean diameter for VLDL particles exhibited the strongest associations with GIMA (meta-*P* value = 1.56 × 10^–30^) and GIMB (meta-*P* value = 1.83 × 10^–15^), respectively. The GIMA was also associated with omega-3 fatty acid levels (meta-*P* value = 4.1 × 10^–8^) and CRP (meta-*P* value = 5.7 × 10^–5^) while GIMB was not, perhaps due to the subtly difference pathway enrichments for each module (Table 1). Other metabolite measures associated with these two modules include mainly the VLDL and HDL subclass of lipoproteins and fatty acids; due to their large size and heterogeneous composition, however, interpretation of metabolic relationships of GIMA and GIMB is limited.

### Long-term stability of interactions between metabolite measures, immune gene modules, and mQTLs

The 216 individuals in both the DILGOM 2007 and 2014 follow-up allowed investigation of the long-term stability of immunometabolic and mQTL relationships. Across this seven-year period, the eight immune gene coexpression networks were strongly preserved (all preservation statistics’ permutation *P* values <0.001; Additional file 10: Table S9). The metabolite–metabolite correlation structure was also largely consistent between DIGOM07 and DILGOM14 (Additional file 4: Figure S6).

**Fig. 4:**
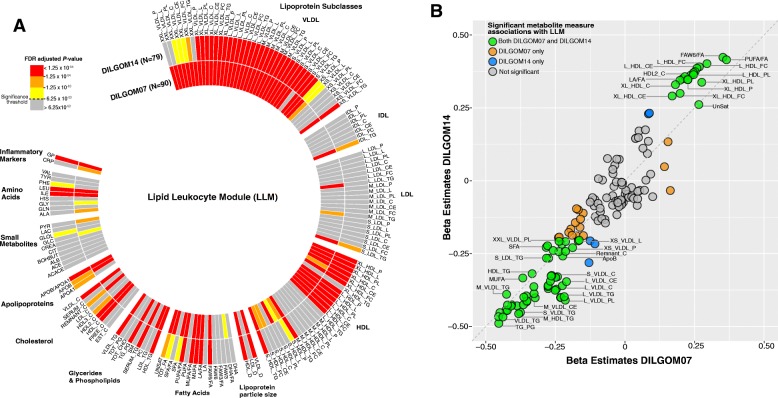
Temporally stable metabolite measure associations with the LLM. **a** Circular heatmap for association between each metabolite measure and the LLM. **b** Comparison of the effect size estimates of metabolite measure association with LLM in DILGOM07 and DILGOM14 shows that the overall association patterns are consistent across the two time-points. Colors denote metabolites that are significantly associated with the LLM in DILGOM07 only (*orange*), DILGOM14 only (*blue*), and across both time-points (*green*). The *grey dashed line* is the x = y line

Next, we examined how metabolite interactions with immune gene modules changed over the 7-year time period (“Methods”). The LLM–metabolite measure associations were the most consistent over time with 90 and 79 metabolite measures reaching significance in DILGOM07 and DILGOM14, respectively, of which 74 were significant at both time points (Fig. 4a; Additional file 11: Table S10). The direction and effect size of LLM–metabolite measure associations were largely maintained (Fig. 4b). For the neutrophil module, the pyruvate association was significantly maintained over time; however, there was some evidence that other expected associations with NM were stable over time, including GlycA (Additional file 11: Table S10). While no associations with metabolite measures were significantly maintained for the platelet module, rs1354034 was a temporally stable mQTL of PM (mQTL *P* value = 4.87 × 10^–7^). No other mQTLs reached significance for temporal stability.

While we were powered to topologically replicate immune modules between time-points, power to detect module–metabolite associations and mQTLs was still limited, with only the strongest associations reaching significance. For the latter, the effect sizes for module associations were generally consistent between the time points (Additional file 4: Figure S7), with the exception of GIMB. With the particularly strong consistency of associations for the LLM and NM, it may be that smaller modules, which capture more defined transcriptional programs, are the most temporally stable in terms of their phenotype associations. However, given the robustness of these associations between independent cohorts, we anticipate that, as long-term omics follow-up of population-based cohorts increase in sample size, more of these discovered associations will become statistically significant over time.

## Conclusions

This study has utilized over 2000 individuals to map the immuno-metabolic crosstalk operating in circulation. We have identified and characterized eight robust immune gene modules, their genetic control, and interactions with diverse metabolite measures, including many of clinical significance (e.g., triglycerides, HDL, LDL, branched-chain amino acids). Also, several significant metabolite measures identified here, particularly branched chain amino acids and fatty acids, have been previously shown to be predictive of cardiovascular events and the development of T2D [6, 50]. Furthermore, our findings are consistent with and build upon those of previous studies. In addition to five newly identified gene modules, their mQTLs and metabolite interactions, we have replicated the previously characterized LL module and confirm its association with lipoprotein subclasses, lipids, fatty acids, and amino acids [13, 14]. Associations between the core genes in the LL module and isoleucine, leucine, and various lipids were also identified independently in the KORA cohort [12]. Importantly, we have shown the long-term stability of LL and neutrophil module coexpression and interactions with metabolite measures, and we have greatly expanded the number of known biomarkers associated with the NM from one (GlycA) to 123 [10]. Our study has also expanded the widespread *trans* eQTL effects at the *ARHGEF3* locus [23], shows them to be strongly maintained within individuals over time, and further identifies extensive interactions with lipoprotein measures that may be a consequence of these *trans* effects.

Taken together, our analyses illustrate the rapidly growing body of evidence intimately linking the immunoinflammatory response to the blood metabolome. With finer-resolution maps of these interactions, new biomarkers of chronic and acute inflammatory states are likely to emerge. With in vivo and interventional studies, modulation of these metabolite–immune interactions through existing lipid-lowering medications, gut microbe effects, or dietary changes may provide new ways the immune system itself can be utilized to lessen the burden of cardiometabolic disease.

## Methods

### Study populations

This study used data from two population-based cohorts, the Dietary, Lifestyle, and Genetic determinant of Obesity and Metabolic syndrome (DILGOM; N = 518) and the Cardiovascular Risk in Young Finns Study (YFS; N = 1650), which have been described in detail elsewhere [13, 51]. All subjects enrolled in these studies gave written informed consent.

The DILGOM study is a subsample of the FINRISK 2007 cross-sectional population-based survey, which recruited a random sample of 10,000 individuals between 25 and 74 years of age, stratified by sex and 10-year age groups, from five study areas in Finland. All 6258 individuals who participated in the FINRISK 2007 baseline health examination were invited to attend the DILGOM study (N = 5024), 630 of whom underwent at least one of the genotyping, transcriptomics, or metabolomics profiling considered here. In 2014, a follow-up study was conducted, for which 3735 individuals from the original study re-participated. Samples collected in 2007 and 2014 are referred to as DILGOM07 and DILGOM14, respectively.

The YFS is a longitudinal prospective cohort study that started in 1980, with follow-up studies carried out every 3 years, to monitor cardiovascular disease risk factors in children and adolescents from five major regions of Finland (Helsinki, Kuopio, Turku, Oulu, and Tampere). A total of 3596 children and adolescents in age groups 3, 6, 9, 12, 15, and 18 years participated in the baseline study; these children were randomly selected from the national public register and their details are described in [51]. In this current study, data collected from the 2011 follow-up study (participants aged 34, 37, 40, 43, 46, and 49 years) were analyzed.

### Sample collection

Venous blood was collected following an overnight fast in all three studies. Samples were centrifuged and the resulting plasma and serum samples were aliquoted into separate tubes and stored at −70 °C for analyses. Protocols for the blood sampling, physiological measurements, and clinical survey questions were similar across the YFS and DILGOM studies and are described extensively in [13, 52].

### Genotyping and imputation

Whole blood genomic DNA obtained from both cohorts was genotyped using the Illumina 610-Quad SNP array for DILGOM07 (N = 555) [13] and a custom generated 670 K Illumina BeadChip array for YFS (N = 2443) [53]. The 670 K array shares 562,643 SNPs with the 610-quad array. The 670 K array removes poorly performing SNPs from the 610-quad array and improves copy number variation coverage [53]. Genotype calling was performed with the Illuminus clustering algorithm [54]. Quality control was as previously described in [13] and [53] for DILGOM and YFS, respectively. Genotypes were imputed to the 1000 Genomes Phase 1 version 3 reference panel using IMPUTE2 in both DILGOM and YFS [17]. Poorly imputed SNPs based on low call-rate (<0.90 for DILGOM, <0.95 for YFS), low-information score (<0.4), minor allele frequency <1%, and deviation from Hardy–Weinberg equilibrium (*P* < 5 × 10^–6^) were then removed. A total of 7,263,701 SNPs in DILGOM and 6,721,082 in YFS passed quality control, with 6,485,973 common between the two. A total of N = 518 samples in DILGOM and N = 2443 samples in YFS individuals passed quality control filters.

### Metabolomics profiling

Metabolite concentrations for DILGOM07 (N = 4816), DILGOM14 (N = 1273), and YFS (N = 2046) were quantified from serum samples utilizing a high-throughput NMR metabolomics platform (Brainshake Ltd, Helsinki, Finland) [18, 55]. Details of the experimental protocol, including sample preparation, NMR spectroscopy, and metabolite identification, have been previously described in [13, 18]. A total of 159 metabolite measures were assessed, of which 148 were directly measured and 11 were derived (Additional file 1: Table S1). The 148 measures include the constituents of 14 lipoprotein subclasses (98 measurements total), sizes of three lipoprotein particles, two apolipoproteins, eight fatty acids, eight glycerides and phospholipids, nine cholesterols, nine amino acids, one inflammatory marker, and ten small molecules (involved in glycolysis, citric acid cycle, and urea cycle). The lipoprotein subclasses are classified according to size as follows: chylomicrons and extremely large VLDL particles (average particle diameter at least 75 nm); five VLDL subclasses—very large VLDL (average particle diameter of 64.0 nm), large VLDL (53.6 nm), medium VLDL (44.5 nm), small VLDL (36.8 nm), and very small VLDL (31.3 nm); intermediate-density lipoprotein (IDL; 28.6 nm); three LDL subclasses—large LDL (25.5 nm), medium LDL (23.0 nm), and small LDL (18.7 nm); and four HDL subclasses—very large HDL (14.3 nm), large HDL (12.1 nm), medium HDL (10.9 nm), and small HDL (8.7 nm). Measurements with very low concentration, set as zero by the NMR pipeline, were set to the minimum value of that particular metabolite measure. Measurements rejected by automatic quality control or with detected irregularities were treated as missing. Undefined derived ratios arising from measurements with very low concentration (i.e., zero) were also treated as missing. Measurements were log2 transformed to approximate a normal distribution.

CRP, an inflammatory marker, was quantified from serum using a high sensitivity latex turbidimetric immunoassay kit (CRP-UL assay, Wako Chemicals, Neuss, Germany) and an automated analyser (Olympus AU400) in DILGOM07 (N = 5000), DILGOM14 (N = 1308), and YFS (N = 2046). CRP levels were log2 transformed.

### Gene expression, processing, and normalization

Transcriptome-wide gene expression levels were quantified by microarrays from peripheral whole blood using similar protocols in all three cohorts, and have been previously described for DILGOM07 [13] and YFS [56]. Stabilized total RNA was obtained from whole blood using a PAXgene Blood RNA System and the protocols recommended by the manufacturer. In DILGOM07, RNA integrity and quantity were evaluated using an Agilent 2100 Bioanalyzer. In YFS, RNA integrity and quantity were evaluated spectrophotometrically using an Eppendorf BioPhotomer and the RNA isolation process was validated using an Agilent RNA 6000 Nano Chip Kit. RNA was hybridized to Illumina HT-12 version 3 BeadChip arrays in DILGOM07 and to Illumina HT-12 version 4 BeadChip arrays in DILGOM14 and YFS.

For DILGOM07, data were preprocessed as described in Inouye et al. [13]. Briefly, for each array the background corrected probes were subjected to quantile normalization at the strip-level. Technical replicates were combined by bead count weighted average and replicates with Pearson correlation coefficient <0.94 or Spearman’s rank correlation coefficient <0.60 were removed. Expression values for each probe were then log2 transformed. For YFS, background corrected probes were subjected to quantile normalization followed by log2 transformation. For DILGOM14, probes matching to the erythrocyte globin components (N = 4) and those that hybridized to multiple locations spanning more than 10 kb (N = 507) were removed. Probes with average bead intensity of 0 were treated as missing. The average bead intensity was then log2 transformed and quantile normalized. A total of 35,425 (for DILGOM07), 36,640 (for DILGOM14), and 37,115 (for YFS) probes passed quality control.

### Gene co-expression network analysis and replication

Gene co-expression network modules were identified in DILGOM07 (N = 518 individuals with gene expression data) as previously described [10] using WGCNA version 1.47 [57, 58] on all probes passing quality control. Briefly, probe co-expression was calculated as the Spearman correlation coefficient between each pair of probes, adjusted for age and sex. The weighted interaction network was calculated as the element-wise absolute co-expression exponentiated to the power 5. This power was selected through the scale-free topology criterion [57], which acts as a penalization procedure to enhance differentiation of signal from noise. Probes were subsequently clustered hierarchically (average linkage method) by topological overlap dissimilarity [57] and modules were detected through dynamic tree cut of the resulting dendrogram with default parameters and a minimum module size of ten probes [59]. Similar modules were merged together in an iterative process in which modules whose eigengenes clustered together below a height of 0.2 were joined. Module eigengenes, representative summary expression profiles, were calculated as the first eigenvector from a principal components analysis of each module’s expression data.

Module reproducibility and longitudinal stability were assessed in YFS (N = 1650 with gene expression data) and DILGOM14 (N = 333 with gene expression data), respectively, using the NetRep R package version 0.30.1 [19]. Briefly, a permutation test (20,000 permutations) of seven module preservation statistics was performed for each module in YFS and DILGOM14 separately. These statistics test the distinguishability and similarity of network features (density and connectivity) for each module in a second dataset [20]. Modules were considered reproducible where permutation *P* values for all seven statistics were <0.001 (Bonferroni correcting for 40 modules) in YFS, and modules were considered longitudinally stable where *P* values were <0.001 for all seven statistics in DILGOM14. Probe co-expression in YFS was calculated as the Spearman correlation coefficient between age- and sex-adjusted expression levels and the weighted interaction network was calculated as the element-wise absolute co-expression exponentiated to the power 4 as previously described [10]. Probe co-expression in DILGOM14 was calculated as the Spearman correlation coefficient between each pair of probes, and the weighted interaction network defined as the element-wise absolute co-expression exponentiated to the power 5.

To filter out genes spuriously clustered into each module by WGCNA, we performed a two-sided permutation test on module membership (Pearson correlation between probe expression and the module eigengene) for each reproducible module in DILGOM07 and YFS. Here, the null hypothesis was, for each module, that its probes did not truly coexpress with the module. The null distribution of module membership for each module was empirically generated by calculating the membership between all non-module genes and the module’s eigengene. *P* values for each probe were then calculated using the following permutation test *P* value estimator [60]:

$$ p=\frac{b+1}{\mathrm{v} + 1}-{\displaystyle {\int}_0^{\raisebox{1ex}{$0.5$}\!\left/ \!\raisebox{-1ex}{${v}_t+1$}\right.}F\left(b;\mathrm{v},{v}_t\right)d{v}_t} $$

where *b* is taken as the number of non-module genes with a membership smaller or greater than the test gene’s module membership, whichever number is smaller; *v*, the number of permutations calculated, and *v*_*t*_, the total number of possible permutations, are both the number of non-module genes. The resulting *P* value was multiplied by 2 because the test was two-sided. To adjust for multiple testing, FDR correction was applied to the *P* values separately for each module using the Benjamini and Hochberg method [61]. We rejected the null hypothesis at FDR adjusted *P* value <0.05 in both DILGOM07 and YFS, deriving a subset of core probes for each module.

### Functional annotation of immune modules

Immune modules were identified through over-representation analysis of Gene Ontology (GO) terms in the core gene set for each of the 20 reproducible modules using the web-based tool GOrilla [21] with default parameters (performed March 2016). GOrilla was run on two unranked gene lists where core module genes were given as the target list and the background list was given as the 25,233 human RefSeq genes corresponding to any probe(s) passing quality control in both DILGOM07 and YFS. A hypergeometric test was calculated to test whether each module was significantly enriched for genes annotated for each GO term in the “biological process” ontology. A GO term was considered significantly over-represented in a module where its FDR corrected *P* value was <0.05. FDR correction was applied in each module separately. Significant GO terms for each module were further summarized into a subset of representative GO terms with REVIGO [22] using the RELSIM semantic similarity measure and a similarity cut-off value *C* = 0.5 on genes from *Homo sapiens*. A module was considered to be immune-linked where the representative GO term list contained the parent GO term GO:0002376 (immune system process) and/or GO:0002682 (regulation of immune system processes). We further performed a literature-based search for genes in the respective modules. Module names, which were assigned based on both GO enrichments and literature-based searches, indicate the likely immune-related processes the modules might be involved in; there’s no implication of exclusivity or that this is the only set of genes involved in that particular process.

### Statistical analyses

Reproducible associations between modules and metabolite measures were identified through linear regression of each immune module eigengene on each of the 159 metabolite measures and CRP in both DILGOM07 and YFS. Prior to analysis, metabolite data were first subsetted to individuals with matching gene expression profiles, followed by removal of subjects on cholesterol lowering drugs, for YFS (N = 62) and DILGOM07 (N = 74). Pregnant women in YFS (N = 10) and DILGOM (N = 2) were further removed from the analysis. A total of 440 individuals in DILGOM07 and 1575 individuals in YFS had matched gene expression and metabolite data, excluding pregnant women and those individuals taking lipid-lowering medication. Models were adjusted for age, sex, and use of combined oral contraceptive pills. Module eigengenes and metabolite measures were scaled to standard deviation units. To maximize statistical power, a meta-analysis was performed on the DILGOM07 and YFS associations using the fixed-effects inverse variance method implemented in the “meta” R package (https://cran.r-project.org/web/packages/meta/index.html). The meta-*P* values for the 160 metabolite measure associations (including CRP) within each module were FDR corrected using the widely used Benjamini–Hochberg procedure [61]. An association was considered significant at FDR adjusted *P* value <6.25 × 10^–3^ (0.05/8 modules). This Bonferroni-adjusted threshold was chosen to further adjust for the multiple modules being tested. To assess the potential confounding effects of blood cell type abundance on module metabolite measure association, the model was rerun in YFS adjusting for leukocyte (for CCLM, VRM, BCM, NM, LLM, GIMA, GIMB) and platelet (for PM) counts available for this cohort. The beta values and *P* values generated with and without adjusting for cell count were then compared. Additionally, to assess the possible effect of cell counts on expression profiles, cell counts were associated with module eigengenes.

Associations between modules and metabolite measures were tested for longitudinal stability in DILGOM14 using a linear regression model of each immune module eigengene on each of the 159 metabolite measures and CRP. A total of 216 individuals in DILGOM had matched gene expression and metabolite data in both 2007 and 2014, after removing pregnant women and individuals on lipid lowering medication at either time point (N = 70). Models were adjusted for age and sex. Information on use of oral contraceptives was not available for this cohort. It is worth noting that >60% of women were more than 50 years old; hence, we would expect that rates of contraceptive use would be low and therefore not a significant confounder. Module eigengenes and metabolite measures were scaled to standard deviation units. An association was considered longitudinally stable where the association was significant (FDR adjusted *P* value <6.25 × 10^–3^) in both DILGOM14 and DILGOM07. For sensitivity analysis, the model in DILGOM07 was run without adjusting for oral contraceptive use and this did not affect the significant associations maintained over the two time-points.

Module quantitative trait loci (mQTLs) were identified through genome-wide association scans with each immune module eigengene using the PLINK2 version 1.90 software (https://www.cog-genomics.org/plink2) [62] in DILGOM07 and YFS. A total of 518 individuals had matched gene expression and genotype data in DILGOM07 and 1400 individuals had matched gene expression and genotype data in YFS. Associations were tested using a linear regression model of each eigengene on the minor allele dosage (additive model) of each SNP. Models were adjusted for age, sex, and the first ten genetic principal components (PCs). Genetic PCs were generated from a linkage-disequilibrium (LD) pruned set of approximately 200,000 SNPs using flashpca [63]. *P* values for each association in DILGOM07 and YFS were combined in a meta-analysis using the METAL software [64], which implements a sample size weighted Z-score method. A SNP was considered an mQTL if meta-analysis *P* value (meta-*P* value) was <5 × 10^–8^. Blood cell count data available for YFS were utilized to test the robustness of module associations with mQTLs, where the same model was run with and without adjusting for leukocyte and platelet cell counts.

Significant mQTLs were subsequently tested as expression quantitative trait loci (eQTLs) for genes within their respective modules using Matrix eQTL in both DILGOM07 and YFS [65]. Both *cis* (mQTL within 1 Mb of a given probe) and *trans* (mQTL greater than 5 Mb from a given probe or on a different chromosome) associations were tested. Associations were tested using a linear regression model of probe expression on minor allele dosage (additive model) of the mQTL. Models were adjusted for age, sex, and the first ten genetic PCs. For *trans*-eQTL associations *P* values in DILGOM07 and YFS were combined in a meta-analysis using the weighted Z-score method and considered significant where the meta-*P* value <5 × 10^–8^. For *cis-*eQTL associations, permutation tests were performed in which gene expression sample labels were shuffled 10,000 times to compute an empirical *P* value. The permuted model *P* values and nominal *P* value were combined across DILGOM and YFS07 in meta-analyses using the weighted Z-score method when computing the permutation test *P* value. An mQTL was considered a *cis*-eQTL where the permutation test *P* value <0.05.

## Additional files

Additional file 1: Table S1. List of 159 NMR-based metabolite measures analyzed in this study. (XLSX 34 kb)

Additional file 2: Table S2. Module preservation statistics of gene networks. (XLSX 47 kb)

Additional file 3: Table S3. Replicated modules and their core genes. (XLSX 442 kb)

Additional file 4: All Supplementary methods and Figures S1–S7. (DOCX 2445 kb)

Additional file 5: Table S4. Module pathway enrichments (XLSX 124 kb)

Additional file 6: Table S5. Association between immune modules and blood cell counts (leukocyte and platelet counts). (XLSX 45 kb)

Additional file 7: Table S6. Module–metabolite measure associations (XLSX 190 kb)

Additional file 8: Table S7. Metabolite measures associated with modules, meta-analysis of DILGOM07 and YFS. (XLSX 232 kb)

Additional file 9: Table S8. Module *trans* eQTLs. (XLSX 258 kb)

Additional file 10: Table S9. Module preservation statistics in DILGOM14. (XLSX 50 kb)

Additional file 11: Table S10. Associations of metabolite measures and modules in DILGOM07 and DILGOM14. (XLSX 223 kb)
