# Supplementary material for: An interaction map of circulating metabolites, immune gene networks, and their genetic regulation
Source: Genome Biol. 2017 Aug 1;18:146. doi: 10.1186/s13059-017-1279-y (PMC5540552; doi:10.1186/s13059-017-1279-y)
Supplement: Supplementary file 4 — All Supplementary methods and Figures S1–S7. (DOCX 2445 kb) [file 13059_2017_1279_MOESM4_ESM.docx]

### Supplementary Text

**Figures**

**
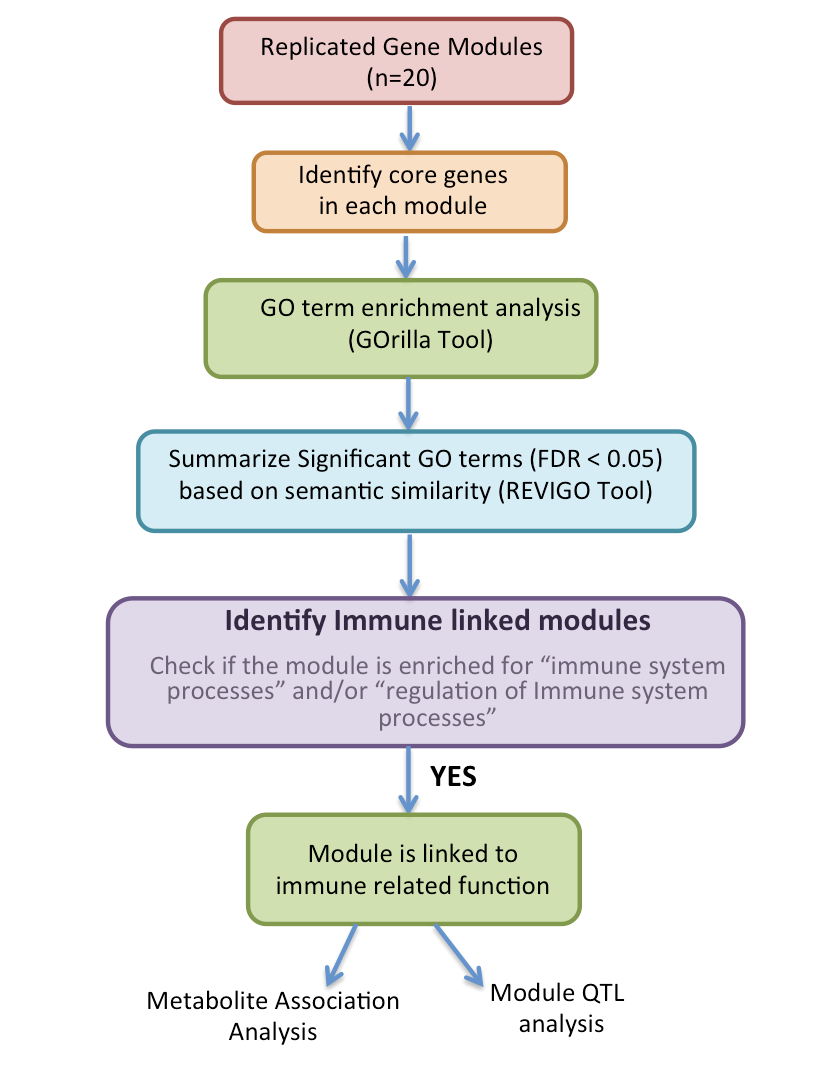
**

**Figure S1: Flowchart representing the steps employed for functional enrichment analysis** of core genes in each of the 20 replicated modules to identify immune-related gene networks.

**
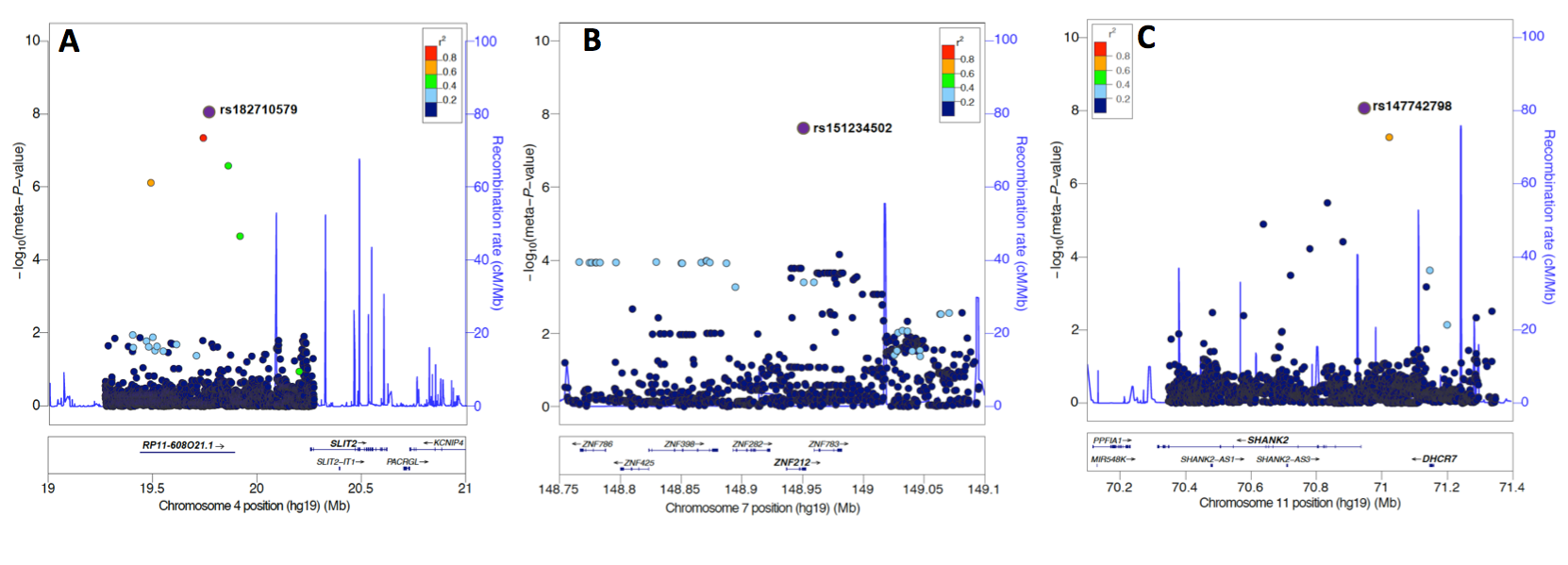
Figure S2: Regional plots of the mQTLs associated with the viral response module (VRM)** at the **(A)** 4p15.31, **(B)** 7q36.1, and **(C)** 11q13.4 regions. For each plot, the circles represent the -log_10_ meta-analysed *P* values (y-axis) of SNPs plotted against their chromosomal position (x-axis). The lead mQTL (rsID) in each plot is denoted by a purple circle, and its pairwise LD (r^2^) strength with other SNPs in the region, estimated from the “1000 genomes Mar 2012 EUR” population, is indicated by color. The blue lines indicate the recombination rates. The plots were generated using the LocusZoom online tool (<http://locuszoom.sph.umich.edu/locuszoom/>).

**
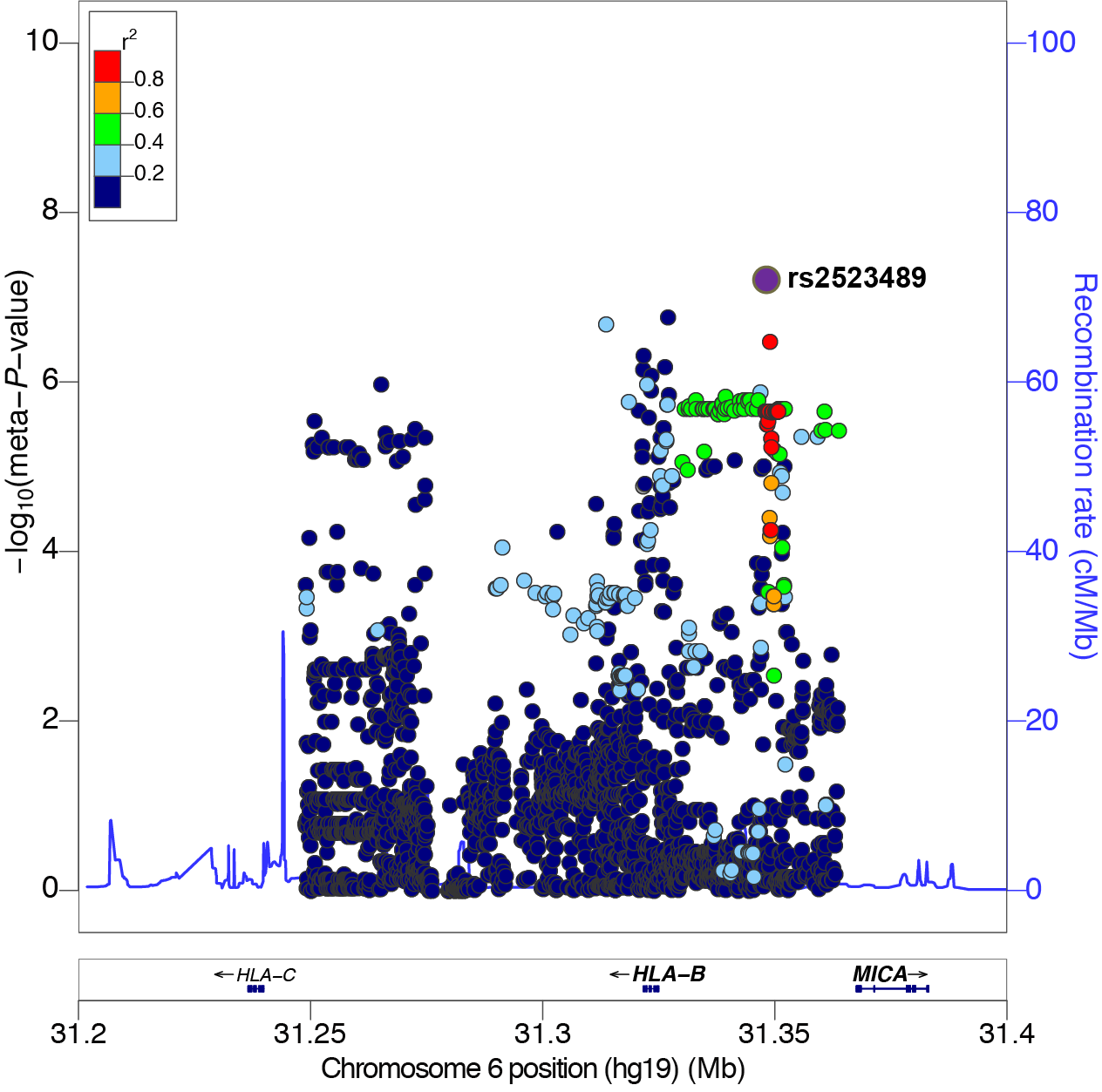
**

**Figure S3:** Regional plot of the mQTL associated with the B cell activity module at the 6p21.33 (HLA) region. The circle represents the -log_10_ meta-analysed *P* values (y-axis) of SNPs plotted against their chromosomal position (x-axis). mQTL (rsID) is denoted by a purple circle, and its pairwise LD (r^2^) strength with other SNPs in the region, estimated from the “1000 genomes Mar 2012 EUR” population, is indicated by color. The blue lines indicate the recombination rates. The plots were generated using the LocusZoom online tool (<http://locuszoom.sph.umich.edu/locuszoom/>).

**
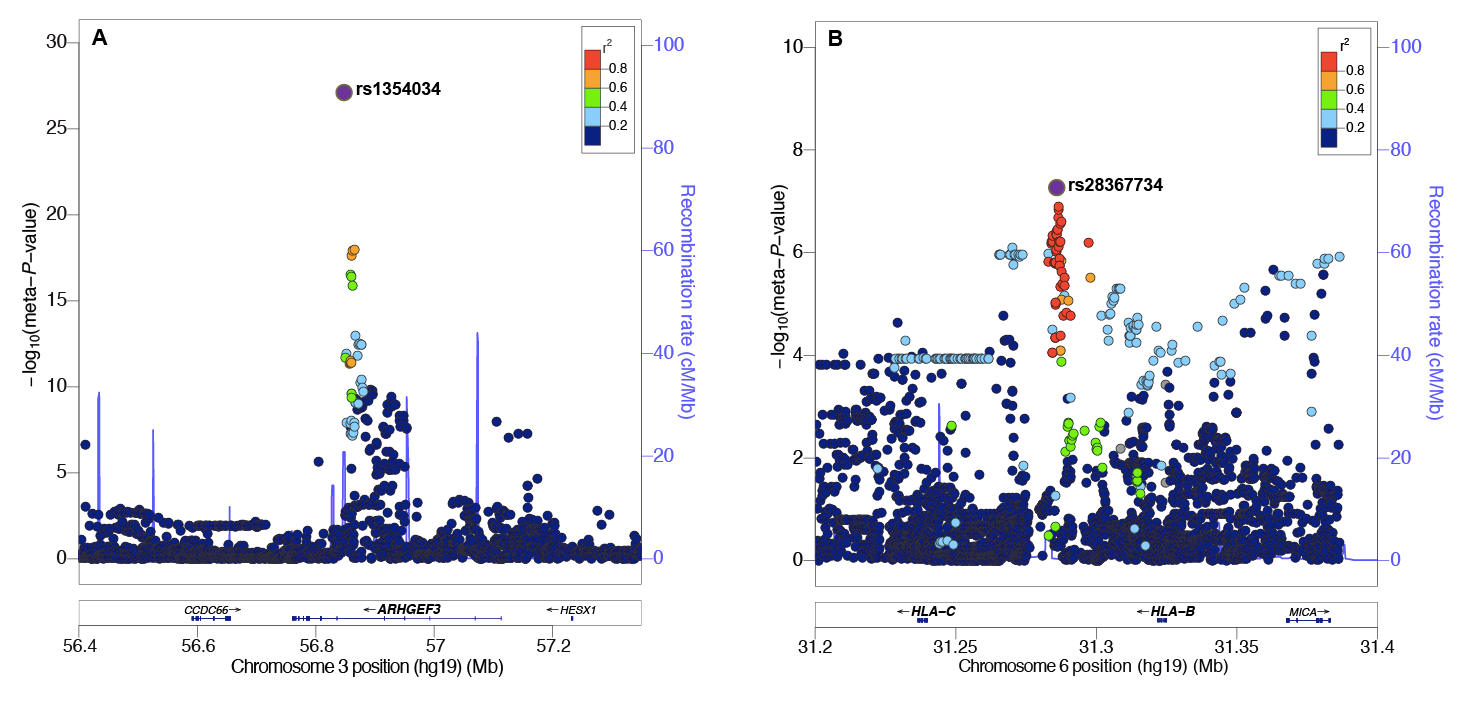
**

**Figure S4: Regional plots of the mQTLs associated with the platelet module (PM)** at regions **(A)** 3p14.3 and **(B)** 6p21.33. For each plot, the circles represent the -log_10_ meta-analysed *P* values (y-axis) of SNPs plotted against their chromosomal position (x-axis). The mQTL (rsID) in each plot is denoted by a purple circle, and its pairwise LD (r^2^) strength with other SNPs in the region, estimated from the “1000 genomes Mar 2012 EUR” population, is indicated by color. The blue lines indicate the recombination rates. The plots were generated using the LocusZoom online tool (<http://locuszoom.sph.umich.edu/locuszoom/>).

**
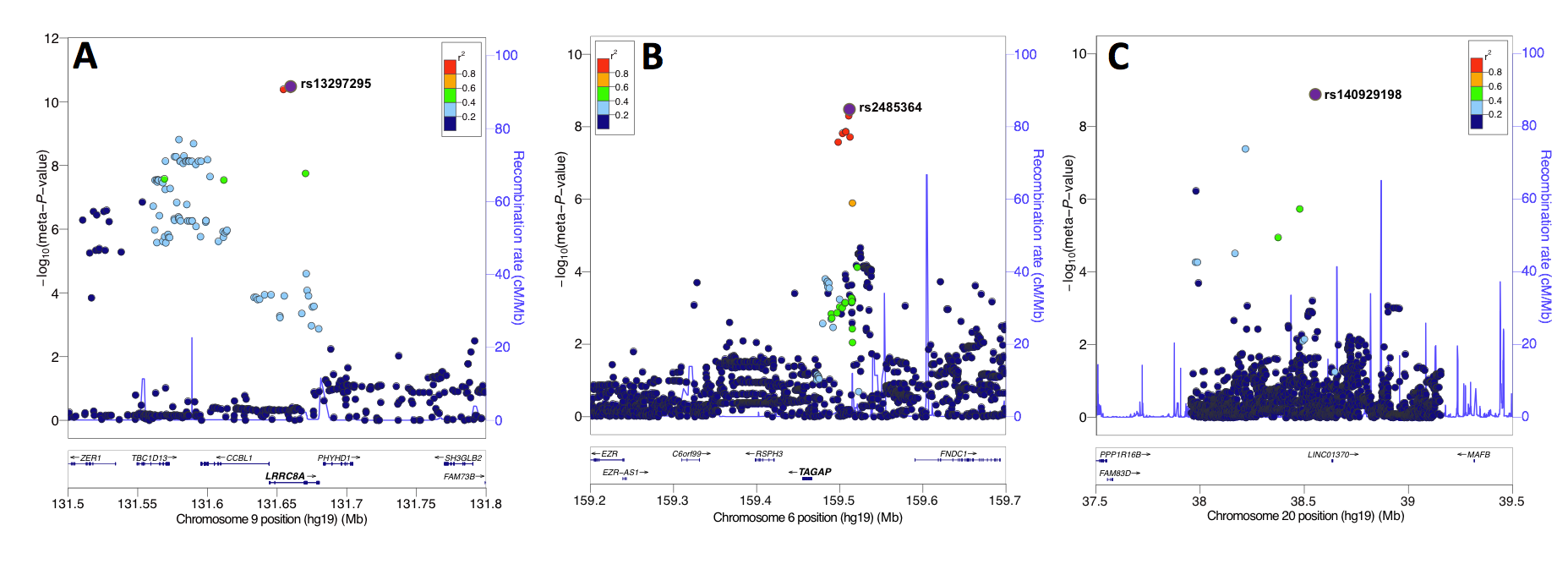
**

**Figure S5: Regional plots of the mQTLs associated with the neutrophil module (NM)** at the **(A)** 9q34.11, **(B)** 6p25, and **(C)** 20q12 regions. For each plot, the circles represent the -log_10_ meta-analysed *P* values (y-axis) of SNPs plotted against their chromosomal position (x-axis). The lead mQTL (rsID) in each plot is denoted by a purple circle, and its pairwise LD (r^2^) strength with other SNPs in the region, estimated from the “1000 genomes Mar 2012 EUR” population, is indicated by color. The blue lines indicate the recombination rates. The plots were generated using the LocusZoom online tool (<http://locuszoom.sph.umich.edu/locuszoom/>).

###
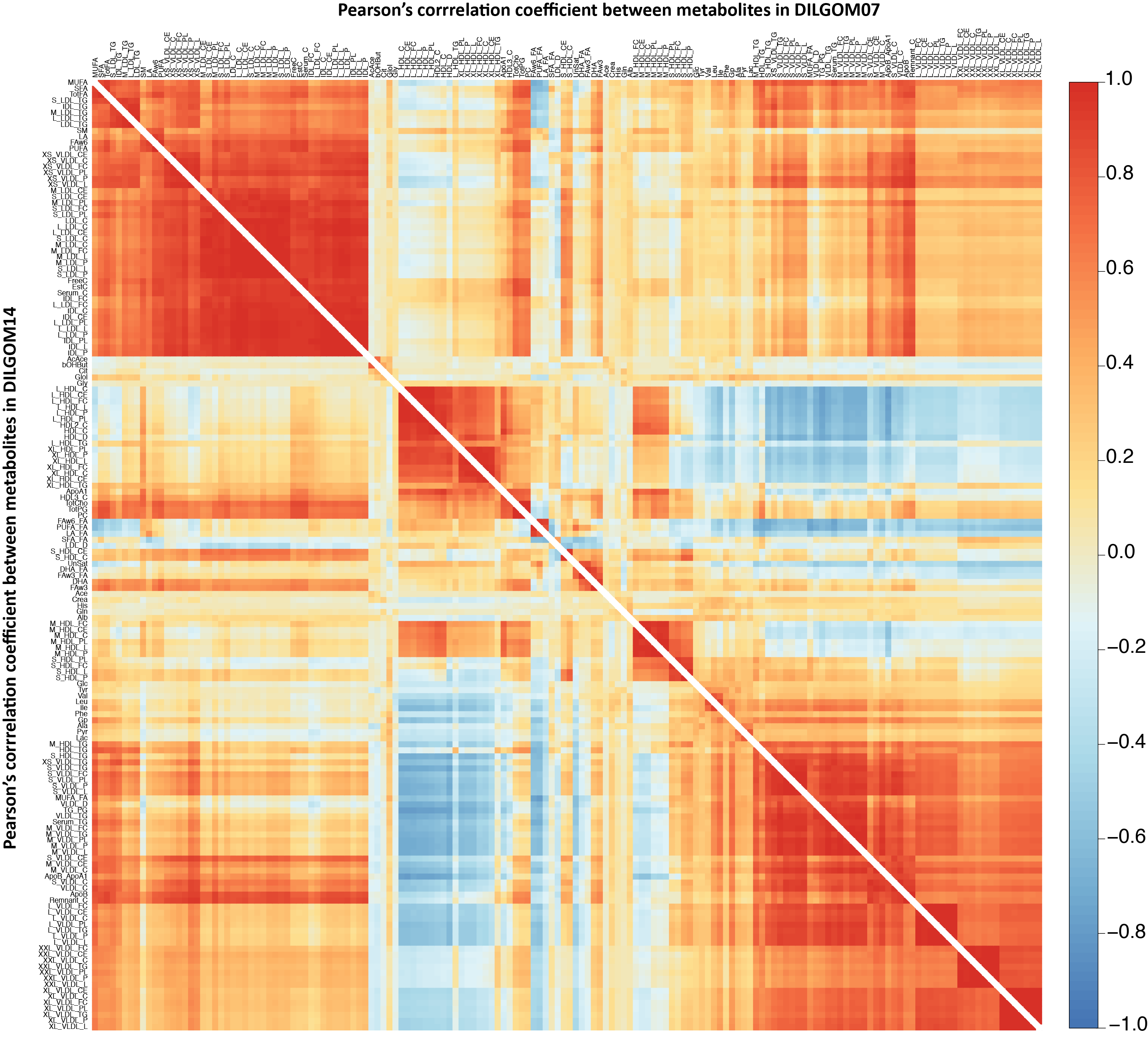


### Figure S6: Heatmap comparing the correlations between metabolites in DILOM07 with those in DILGOM14.Comparison of the correlations between the 158 metabolites within DILGOM07 (upper triangle) with those in DILGOM14 (lower triangle). Each square in each triangle represents the Pearson’s correlation coefficient calculated between the metabolites within each cohort separately. The correlation matrix in DILGOM07 was hierarchically clustered using distance as, 1-absolute value of the correlations. The ordering of rows and columns in DILGOM2014 (lower triangle) was based on DILGOM07. Red and blue indicates positive and negative correlations, respectively.

**
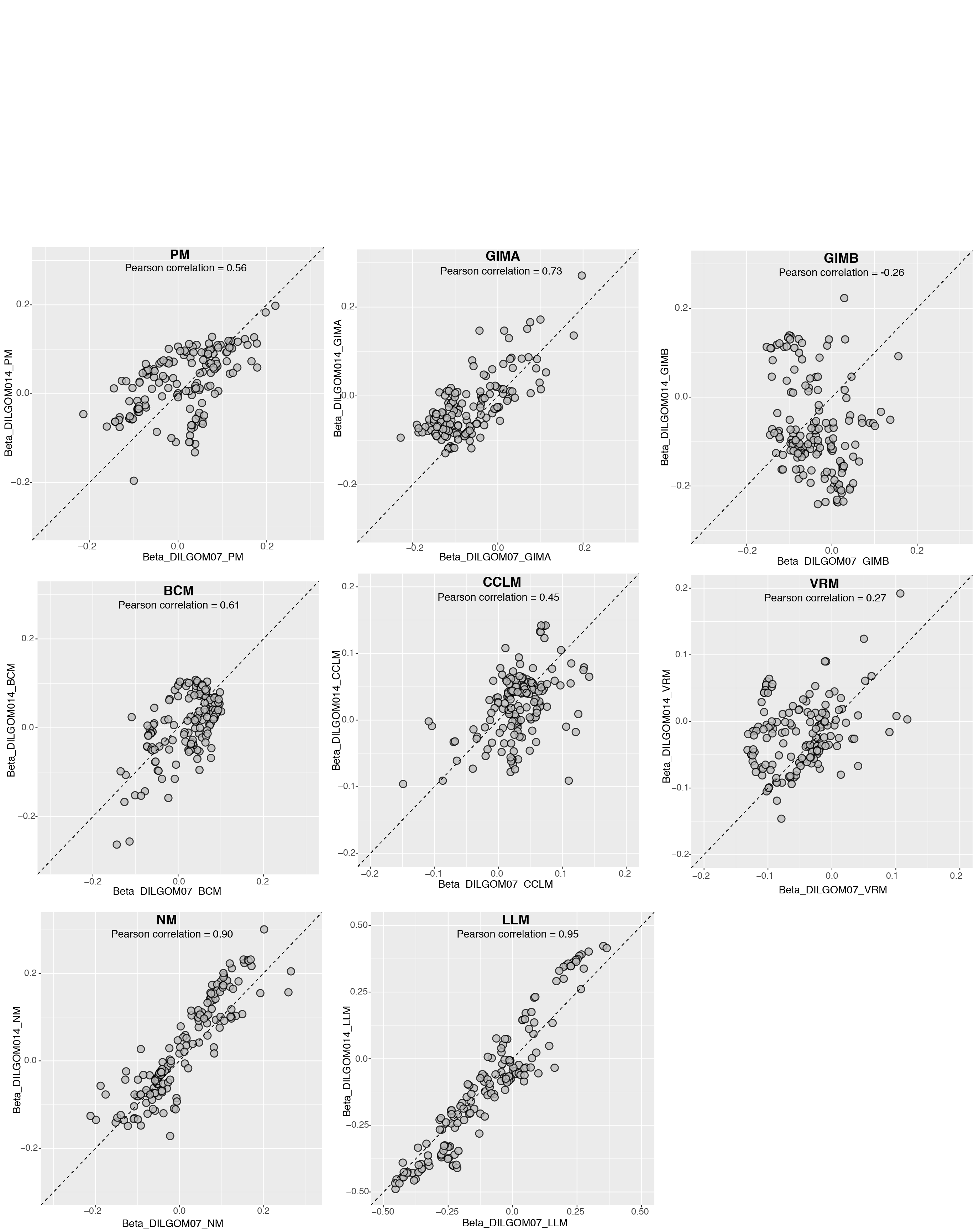
**

**Figure S7: Comparison of the of beta estimates** obtained from the association analysis between metabolites and each immune module in DILGOM07 (x-axis) and DILGOM14 (y-axis). The Pearson’s correlation coefficient (r) is shown on the top of each plot.
